# Editorial: The Benefits of Nature-Based Solutions to Psychological Health

**DOI:** 10.3389/fpsyg.2021.646627

**Published:** 2021-02-04

**Authors:** Giuseppina Spano, Payam Dadvand, Giovanni Sanesi

**Affiliations:** ^1^Department of Agricultural and Environmental Sciences, University of Bari Aldo Moro, Bari, Italy; ^2^ISGlobal, Barcelona, Spain; ^3^Centro de Investigación Biomédica en Red Epidemiología y Salud Pública (CIBERESP), Madrid, Spain

**Keywords:** psychological health, mental health, well-being, human-environment interaction, urban green space, natural environment

## Introduction

Nature-based solutions (NBS) have been defined by the European Commission as actions aiming to provide environmental, social, and economic benefits through the inclusion of natural features in the urban environment. The exposure to natural environments, including NBS in urban contexts, has been associated with a large number of health benefits (Ulrich et al., 1991; Berman et al., 2008; Spano et al., 2020), particularly mental health and well-being among those most studied. Earlier studies on such benefits have been mainly experimental, investigating the short-term effects of brief exposure to natural environments on stress reduction and cognitive restoration (Kaplan and Kaplan, 1989; Berto, 2005; Nilsson et al., 2010; Carrus et al., 2017). More recently, large-scale epidemiological studies have provided further evidence of the long-term effects of sustained exposure to green spaces on mental health and well-being throughout the life course (Hartig et al., 2014; Gascon et al., 2015; McCormick, 2017; de Keijzer et al., 2020).

Several dimensions characterize the human–nature interaction. In this sense, the present Research Topic was intended to provide an overview of studies focusing on the association of exposure to natural environments in urban, peri-urban, and rural settings with psychological well-being and mental health from different perspectives.

## Effects During Childhood, Adolescence and Young Adulthood

Touloumakos and Barrable offered an interesting perspective on the potential protective effect of nature engagement in children with adverse childhood experiences such as family abuse and dysfunctional experiences related to poor parenting skills. These childhood experiences have been shown to produce physiological and psychological symptoms, including chronic stress, cognitive dysfunctions, psychopathologies, and cardiovascular and metabolic disorders. From an overview of published studies, a significant gap emerged in the potential therapeutic and protective effect of nature engagement (NE) in individuals with adverse childhood experiences. In this perspective, the authors suggested that NE can positively impact the physiological and psychological health of children who have experienced trauma, opening the door to the potential beneficial effects of nature inducing a retroactive effect.

Inconsistent evidence has been found on the association between green space and pro-social behavior in children and adolescents. Putra et al. presented a systematic review on 15 available studies highlighting mixed findings and methodological heterogeneity. In particular, green space indicators and pro-social behavior measures varied among studies and a lack of mediators and potential confounding variables was detected. This review provides preliminary evidence on the association between green space and pro-social behavior. Nevertheless, the authors underlined the need to further develop rigorous studies in order to identify underlying pathways in this promising association and suggested considering the role of perceived quality of green space as an important variable in relation to pro-social behavior.

Another neglected topic was found to be the relationship between NE and pro-social behavior among undergraduate students, a sub-population that is well-known for being at high risk of stress, anxiety and depression. Sachs et al. showed that NE during childhood appeared to be positively associated with NE during college. Similarly, a pro-environmental attitude was positively associated with NE both in childhood and during college. This work enhanced the long-term effect of green space exposure during childhood also in light of the considerable decrease of time spent in nature during college. However, their study did not reveal any association between NE and stress levels, probably because, as reported by some participants, contact with nature could have been perceived as a waste of time given the many school commitments.

## Effects During Adulthood

Pro-environmental behavior was also investigated in a study by Panno et al. in a sample of urban park visitors. The authors tested a novel modeling framework in which pro-environmental behavior was directly predicted by an emotion-regulating strategy (namely, cognitive reappraisal) and indirectly through the experience of “being away” when embedded in a natural environment. This study offered relevant insights on the role of cognition and perceived restorative experience of the natural environment, which together prove to significantly trigger pro-environmental choices.

Although its multiple benefits are well-known, direct contact with the natural environment is not always possible. For this reason, Browning et al. compared the impacts of simulated (i.e., virtual) and actual nature experiences on mood based on data from six published studies. Simulated experiences varied from looking at pictures to walking on the treadmill while watching a video of the natural environment. Results suggest a greater positive effect on mood due to the real experience of green exposure. This work also highlighted the possibility of considering valid alternatives, especially when green exposure is not possible, as in the case of recent confinement due to the COVID-19 pandemic.

Surprising findings emerged in the study results by Trammell and Aguilar. They found that natural environments could positively affect performance improvement when the required task involves a moderate attention level, while no effect was found on tasks requiring minimal or greater amounts of attention. However, this positive effect was greater in the indoor environment than in the natural environment. Physical activity, on the other hand, seemed to have beneficial effects on affect and cognition regardless of whether it was carried out in indoor or natural environments. This study suggests that a complex relationship exists between the natural environment and the benefits that cannot be reduced to the concepts of exposure and restorativeness. Other mechanisms may play a role in diversifying outcomes, including adaptation mechanisms.

With regard to complexity, new theoretical insights on green space exposure and the reduction of psychosis risk have been advanced by Ebisch. This perspective paper provides an overview of the unexplored role of the self and brain network interactions in the connection between green space and psychosis. This topic is of particular relevance, since psychotic disorders (e.g., schizophrenia) have been a priority for the public health agenda due to the pervasiveness and chronicity of the disorder, high rate of hospitalizations and comorbidities, and premature mortality.

## Conclusions

This Research Topic provides a multidisciplinary perspective of the human–nature interaction throughout the life-course in association with mental health and well-being. The studies included in this topic have generally demonstrated potential beneficial associations; however, they have also highlighted inconsistencies in the evidence available in terms of their applied methodologies and reported findings.

## Author Contributions

All authors listed have made a substantial, direct and intellectual contribution to the work, and approved it for publication.

## Conflict of Interest

The authors declare that the research was conducted in the absence of any commercial or financial relationships that could be construed as a potential conflict of interest.
